# Development and Validation of Nomograms Predicting the 5- and 8-Year Overall and Cancer-Specific Survival of Bladder Cancer Patients Based on SEER Program

**DOI:** 10.3390/jcm12041314

**Published:** 2023-02-07

**Authors:** Peng Wen, Jiao Wen, Xiaolong Huang, Fengze Wang

**Affiliations:** 1 Department of Urology, People’s Hospital of Hechuan Chongqing, Chongqing 401520, China; 2 Xi’an Savaid Obstetrics and Gynecology Hospital, Xi’an 710032, China; 3 Department of Urology, The First Affiliated Hospital of Chongqing Medical University, Chongqing 400016, China; 4 Faculty of Medicine, University of Basel, 4056 Basel, Switzerland

**Keywords:** bladder cancer, overall survival, cancer-specific survival, nomogram, SEER, prognosis

## Abstract

Background: Bladder cancer is often prone to recurrence and metastasis. We sought to construct nomogram models to predict the overall survival (OS) and cancer-specific survival (CSS) of bladder cancer patients. Methods: A reliable random split-sample approach was used to divide patients into two groups: modeling and validation cohorts. Uni-variate and multivariate survival analyses were used to obtain the independent prognostic risk factors based on the modeling cohort. A nomogram was constructed using the R package, “rms”. Harrell’s concordance index (C-index), calibration curves and receiver operating characteristic (ROC) curves were applied to evaluate the discrimination, sensitivity and specificity of the nomograms using the R packages “hmisc”, “rms” and “timeROC”. A decision curve analysis (DCA) was used to evaluate the clinical value of the nomograms via R package “stdca.R”. Results: 10,478 and 10,379 patients were assigned into nomogram modeling and validation cohorts, respectively (split ratio ≈ 1:1). For OS and CSS, the C-index values for internal validation were 0.738 and 0.780, respectively, and the C-index values for external validation were 0.739 and 0.784, respectively. The area under the ROC curve (AUC) values for 5- and 8-year OS and CSS were all greater than 0.7. The calibration curves show that the predicted probability values of 5- and 8-year OS and CSS are close to the actual OS and CSS. The decision curve analysis revealed that the two nomograms have a positive clinical benefit. Conclusion: We successfully constructed two nomograms to forecast OS and CSS for bladder cancer patients. This information can help clinicians conduct prognostic evaluations in an individualized manner and tailor personalized treatment plans.

## 1. Introduction

Bladder cancers are the most common malignancies and rank as the 10th most common cancer according to global statistics [1]. Notably, in 2020, 573,278 cases of bladder cancer were newly diagnosed, and 212,536 patients died of bladder cancer worldwide [1]. Moreover, bladder cancers can easily recur and metastasize, and the 5-year survival rate among bladder cancer patients is less than 62% because 33% of bladder cancers are likely to metastasize at an early stage [2]. Thus, it is imperative to establish a model to predict the prognosis of bladder cancer patients in advance.

Currently, a coherent approach for evaluating these prognoses involves the use of guidelines from the American Joint Committee on Cancer (AJCC) Staging Manual [3]. However, the prognoses of bladder cancer patients are influenced by factors that do not appear in this manual, such as age, sex, race, surgery, radiation, and grade stage [4,5]. Notably, these studies show that metabolic syndrome, waist circumference, and detrusor muscle thickening were also the important risk factors influencing the recurrence and progression of bladder cancer [6,7]. An approach that accounts for additional relevant elements could provide more comprehensive predictions of prognoses than the TNM staging system. The nomogram is constructed by R software based on the prognostic independent risk factors obtained from the Kaplan–Meier and multi-variate Cox proportional hazard survival analysis. Each independent risk factor is assigned a corresponding value in the nomogram and the values are summed to predict the final survival rate [8]. Most importantly, the use of a nomogram for the early detection of prostate cancers is incorporated into the National Comprehensive Cancer Network (NCCN) clinical guidelines [9]. Moreover, nomograms have also been applied for hepatocellular carcinoma [10], buccal cancer [11] and gastric cancer [12]. Nomograms have been established via a random split-sample approach, and many researchers constructed credible models by utilizing a split ratio of 1:1 or 1:2 [13,14,15]. Hence, in this study, we sought to construct credible nomogram models to predict the 5- and 8-year overall survival (OS) and cancer-specific survival (CSS) of bladder cancer patients to provide support to surgeons and conduct a personalized prognosis evaluation.

## 2. Materials and Methods

### 2.1. Patients Information and Survival Analysis

We collected detailed data for 20,857 bladder cancer patients from 2004 to 2012 from the SEER database: http://seer.cancer.gov (accessed on 1 June 2021). The collected data include age, sex, race, grade, surgery, radiation, T stage, N stage, M stage, overall survival (OS), cancer-specific survival (CSS) and survival time. The categories used for race included White, Black and other (American Indian/AK Native and Asian/Pacific Islander). The categories used for the grades were grades I, II, III based on the WHO 1973 classification. Grades I, II, III represent well, moderately, and poorly differentiated bladder cancers, respectively. The duration of OS was the time from the diagnosis to death or the last follow-up time point. However, the CSS is a parameter that mainly focuses on the death due to bladder cancer. We used the SPSS software “random sample of cases” option and entered 50 in the “approximately” option to achieve a 1:1 split-ratio between the modeling group (n = 10,478) and the validation group (n = 10,379). We conducted survival analyses using Kaplan–Meier analysis and multivariate Cox proportional hazard models to identify independent risk factors influencing OS and CSS for bladder cancer patients [16]. Data analysis was conducted using SPSS software (version 21.0, Chicago, IL, USA). Two-sided *p* < 0.05 was regarded as indicative of statistical significance.

### 2.2. Nomogram Model Establishment and Risk Classification

The above independent risk factors were used to establish nomograms to predict 5- and 8-year OS and CSS for patients, using the R package “cmprsk”, transforming the independent prognostic risk factors into a visual graph. In the graph, each score axis is quantitatively scored according to the classification of the factors, e.g., Grade I, II, III had different scores. Finally, the scores of all factors were summed according to the condition of each patient, and the total score can be drawn with vertical lines to the 5-year and 8-year survival rate axes to obtain the final survival rate prediction. In addition, the patients were classified into high-risk and low-risk groups based on cut-off value via R package “maxstat”. Log-rank test was performed on the prognosis of patients in high-risk and low-risk groups, and survival curves were drawn by R package “survminer”.

### 2.3. Nomogram Model Validation

The nomograms were validated by re-bootstrapping 1000 times, applying ten-fold cross-validation measures. The con-concordance index (C-index) and the receiver operating characteristic curve (ROC) were used to assess the nomogram’s discrimination, specificity and sensitivity through “hmisc” and “timeROC” packages in R software [17]. The calibration curves were used to evaluate the actual and predicted outcome via “rms” package in R software. The calibration curves included two lines: the dotted 45-degree ideal line and the actual line. The separation between these two lines indicated the precision of a nomogram model. In addition, decision curves were drawn to reflect the clinical benefit of the predictive nomogram model using “stdca.R” package [18]. 

## 3. Results

### 3.1. Clinicopathological Data for Patients and Survival Analysis

In total, 10,478 and 10,379 bladder cancer patients were included in the nomogram modeling and validation cohorts, respectively, with a split ratio of 1:1. In the modeling cohort, 7768 patients (74.1%) were male, and 9234 patients (88.1%) were white. The main type of lesions was grade III (66.9%). A total of 10,145 patients (96.8%) underwent surgery and 922 patients (8.8%) received radiotherapy. In this cohort, 84.3%, 92.4%, and 95.5% of tumors were in stages T1–T2, N0, and M0, respectively. Detailed clinical data for the validation cohort are presented in Table 1. 

**Figure 1:**
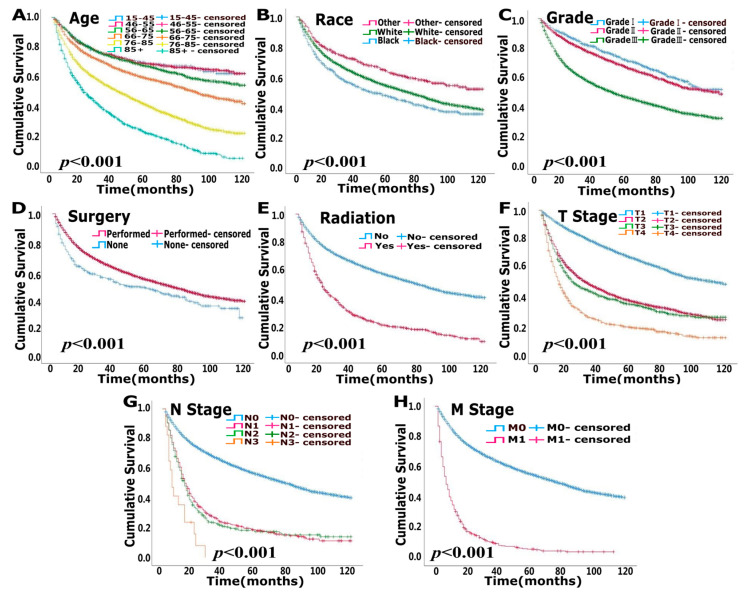
Uni-variate survival analysis of overall survival (OS) in modeling cohort. (**A**). Age (**B**). Race (**C**). Grade (**D**). Surgery (**E**). Radiation (**F**). T stage (**G**). N stage (**H**). M stage.

**Figure 2:**
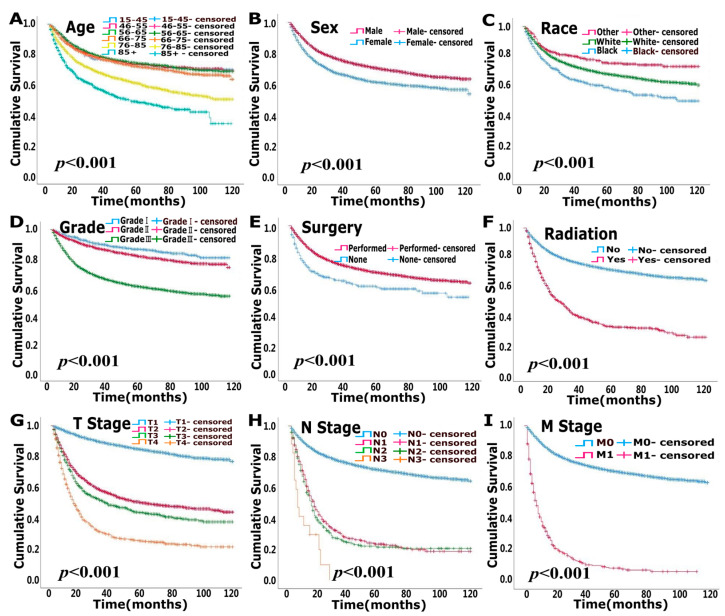
Uni-variate survival analysis of cancer-specific survival (CSS) in modeling cohort. (**A**). Age (**B**). Sex (**C**). Race (**D**). Grade (**E**). Surgery (**F**). Radiation (**G**). T stage (**H**). N stage (**I**). M stage.

The median follow-up times for the modeling and validation cohorts were 37 months (2–119 months) and 37 months (2–119 months), respectively. In total, 5105 patients were deceased at the last date of follow-up; 3125 of these patients died due to bladder cancer, and 1980 patients died of other causes that were not recorded in the SEER database. OS analysis showed that age, race, pathological grade, surgery, radiation, T stage, N stage and M stage were independent risk factors (*p* < 0.05) (Figure 1, Table 2). CSS analysis indicated that age, sex, race, pathological grade, surgery, radiation, T stage, N stage and M stage were independent prognostic elements (*p* < 0.05) (Figure 2, Table 3).

### 3.2. Nomogram Model Establishment and Risk Classification

**Figure 3:**
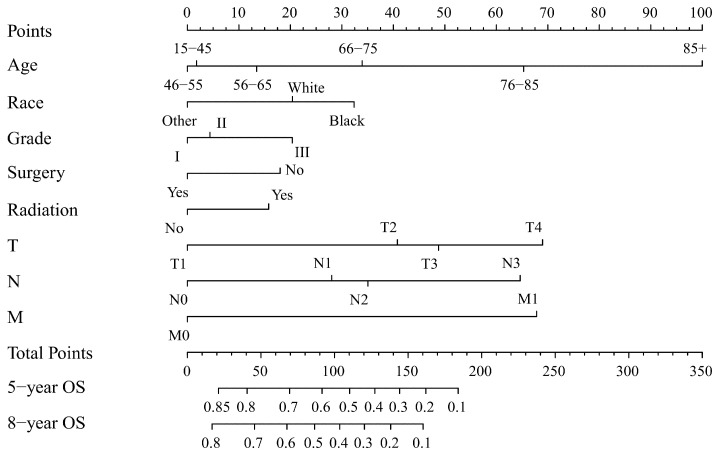
Nomogram forecasting 5-year and 8-year overall survival (OS). Abbreviations: Others: American Indian/Alaska Native/Asian or Pacific Islander. Grade I: Well differentiated. II: Moderately differentiated. III: Poorly differentiated.

**Figure 4:**
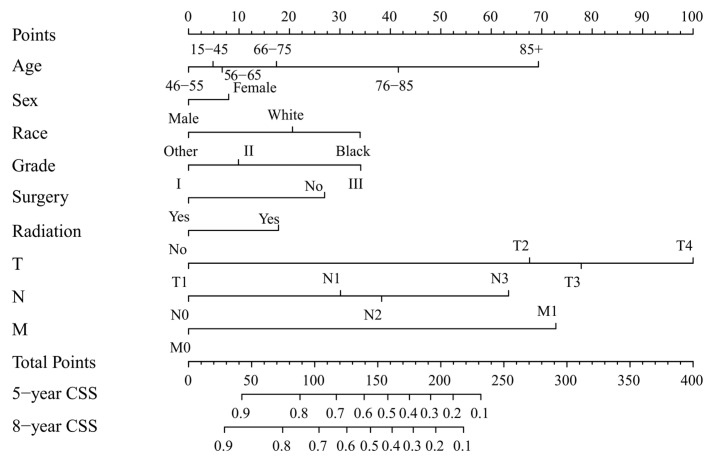
Nomogram forecasting 5-year and 8-year cancer-specific survival (CSS). Abbreviation: Others: American Indian/Alaska Native/Asian or Pacific Islander. Grade I: Well differentiated. II: Moderately differentiated. III: Poorly differentiated.

**Figure 5:**
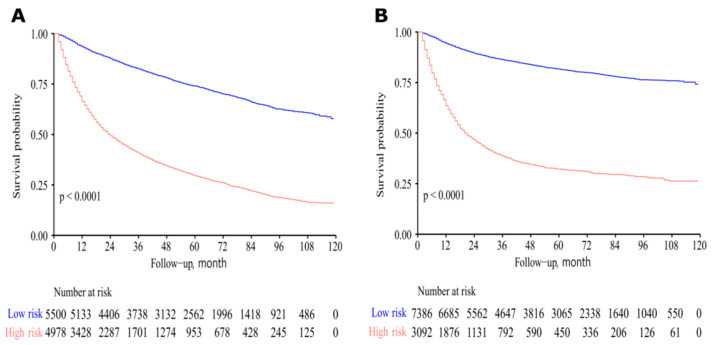
Survival curves of high- and low-risk patients based on nomogram scores. Low-risk patients had improved overall survival (OS) and cancer-specific survival (CSS). (**A**) For OS. (**B**) For CSS.

Two nomograms to predict 5- and 8-year survival were constructed (Figure 3 and Figure 4). Based on the nomograms scores, the OS and CSS cut-off scores to divide patients into low- and high-risk cohorts were 102 and 144, respectively. Patients in the low-risk group had improved OS and CSS compared to high-risk group patients, with statistical significance after the log-rank test (*p* < 0.05) (Figure 5). 

### 3.3. Nomogram Model Validation

**Figure 6:**
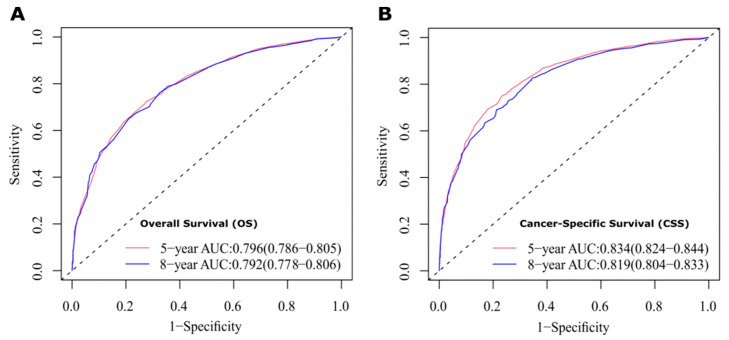
ROC curves to evaluate the sensitivity and specificity of nomograms. (**A**) ROC curves for 5- and 8-year overall survival (OS). (**B**) ROC curves for 5- and 8-year cancer-specific survival (CSS).

**Figure 7:**
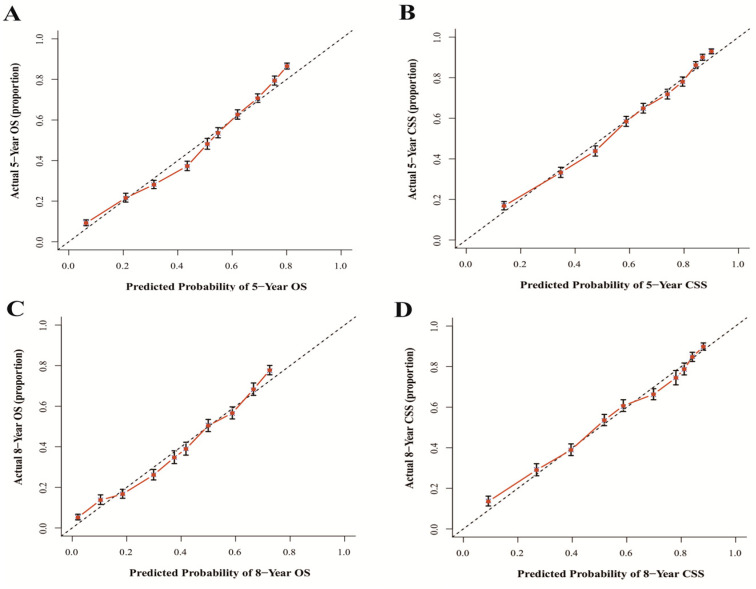
Calibration curves of internal validation for 5- and 8-year OS (**A**,**C**) and 5- and 8-year CSS (**B**,**D**). Abbreviations: The 45-degree dotting line means a nice match between the actual survival (*Y*-axis) and nomogram-forecasted survival (*X*-axis). The vertical line represents 95% confidence intervals.

**Figure 8:**
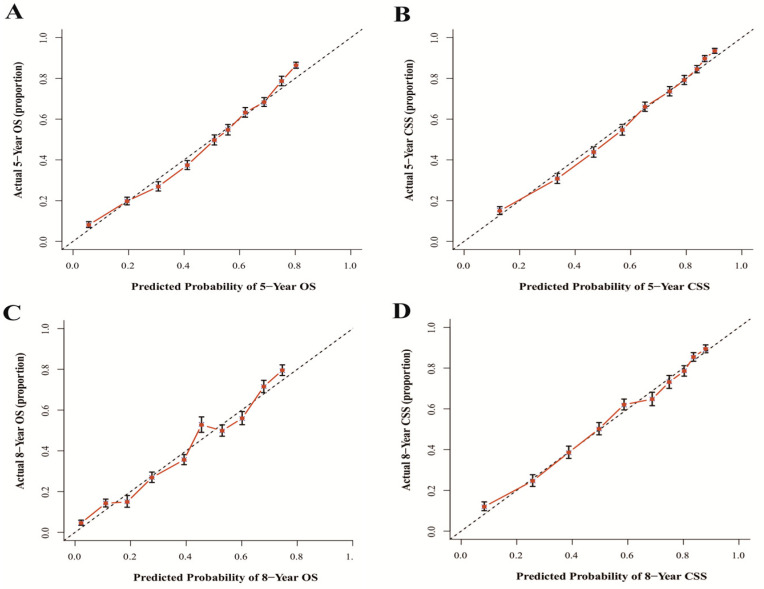
Calibration curves of external validation for 5- and 8-year OS (**A**,**C**) and 5- and 8-year CSS (**B**,**D**). Abbreviations: The 45-degree dotting line means a nice match between the actual survival (*Y*-axis) and nomogram-forecasted survival (*X*-axis). The vertical line represents 95% confidence intervals.

**Figure 9:**
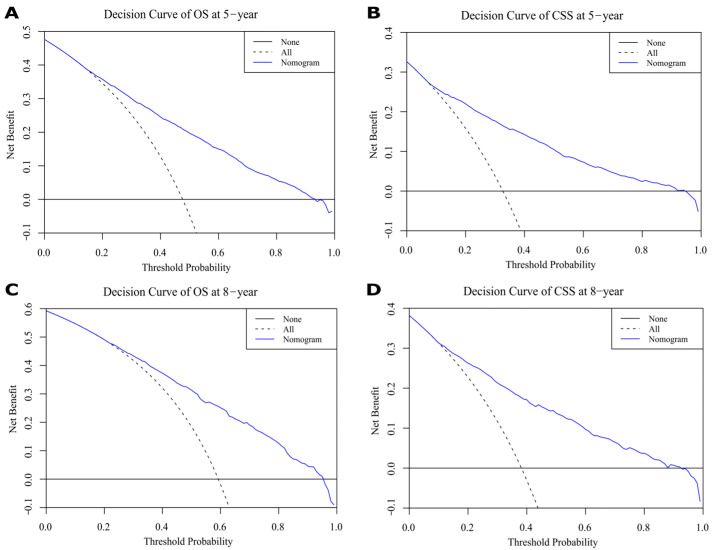
Decision curves for 5- and 8-year overall survival (OS) and cancer-specific survival (CSS) in validation cohort. (**A**,**C**) Decision curves of 5-year OS and CSS. (**B**,**D**) Decision curves of 8-year OS and CSS.

The results show that the C-index values of 0.745 (95% CI: 0.738–0.752) and 0.788 (95% CI: 0.780–0.796) for OS and CSS based on internal validation, respectively. The C-index values for external validation were 0.746 (95% CI: 0.739–0.753) and 0.791 (95% CI: 0.784–0.798) for OS and CSS, respectively. All C-index values for the nomograms were greater than 0.7. According to the results of receiver operating characteristic (ROC) in validation group, the area under the ROC curve (AUC) values for 5- and 8-year OS were 0.796 and 0.792, respectively (Figure 6A). The AUC for 5- and 8-year CSS were 0.834 and 0.819, respectively (Figure 6B). Moreover, the internal and external calibration curves were close to the 45-degree ideal line (Figure 7 and Figure 8). The two nomogram models showed clear clinical benefits in the validation group (Figure 9).

## 4. Discussion

Bladder cancer is a common cancer that originates from bladder cells, covering the inner layer of the bladder. The prevalence and mortality rates in men is 9.5 and 3.3 per 100,000 people, about four times higher than in women globally [1]. The incidence of bladder cancer is highest in southern Europe, western Europe and northern America; thus, this disease is emerging as a public health burden [1]. Bladder cancer is divided into muscle-invasive and non-muscle-invasive types with heterogeneous biology and clinical course according to whether the muscle layer is involved. Non-muscle-invasive bladder cancer is limited to the mucosa and/or only invades the lamina propria [19]. Approximately 80% of bladder cancers are found in the early stages with non-muscle -invasive characteristics and have a high cure rate. Bladder cancer has a 5-year survival rate of approximately 94% with prompt detection and intervention [20]. However, even early-stage bladder cancer can recur after successful treatment. Therefore, patients with bladder cancer usually need to be reviewed after treatment to determine if their cancer has recurred. Evidently, an assessment of the prognosis is vital. Traditionally, estimates of prognosis are based on a population of patients via TNM stages, making personalized evaluation a challenge. To embrace individualized assessment, clinicians need to combine patients’ other information, such as age, sex, pathology grade, radiation and surgery, rather than just TNM stages to empirically predict the outcomes for specific patients [11]. Currently, nomograms are widely used to transform the above clinicopathological parameters into a visualized graph and conveniently predict long-term 5- and 8-year survival [8]. To evaluate accuracy, the concordance index (C-index), receiver operating characteristic (ROC) and calibration curve were applied [21,22,23]. The results show that the two nomograms had a high discrimination (all the C-index > 0.7), high sensitivity and specificity (all the AUC > 0.7). 

Numerous studies show that age, gender and race are important factors that influence the prognoses of bladder cancer [24,25,26]. Epidemiological data show that bladder cancer patients are rarely under 50 years of age [27]. In our study, the majority of patients were older than 55 years, accounting for 87.5%, and the long-term survival rate decreased with age. The underlying reason for this phenomenon might be that elderly patients are vulnerable to treatment-induced toxicity [28]. Bladder cancer is five times more prevalent in men than in women [20]. Based on our data, 74.1% and 25.9% of patients are male and female, respectively. However, we found that females had worse survival than males, and this finding is consistent with the results of other recent studies [29,30]. Research shows that more advanced bladder cancer is more prevalent in female patients than male patients, which is considered the most significant reason for worse OS and CSS [31]. 

In our study, we found that black patients had worse OS and CSS than white or other patients; however, no clear reason for this phenomenon was discovered. Different genetic characteristics, tumor molecular markers, and lifestyles may be associated with the higher incidence of aggressive bladder cancers in black patients [32]. Hence, differences in bladder cancer between races requires further research. In general, surgery and radiotherapy are the main treatments used for curing bladder cancers [24,33]. In our study, overall survival (OS) and cancer-specific survival (CSS) were higher for patients treated with surgery than those who did not undergo surgery. The analysis results demonstrate that OS and CSS were better for bladder cancer patients who did not undergo radiotherapy compared to patients treated with radiotherapy. This is because, in our data, 97% of patients without radiotherapy were treated with surgery, 85.6% at T1–T2 stage, 93.1% at N0 stage, and 96.3% at M0 stage. Moreover, patients who receive radiotherapy are often patients with poor health conditions who cannot tolerate surgery or patients with terminal tumors that have lost their opportunity to have surgery. Currently, in the treatment of bladder cancer, adjuvant radiotherapy continues to be investigated [34]. A multi-center randomized controlled trial of 210 patients with T1 stage, Grade III, Nx and M0 at 37 centers found no statistical difference in 5-year progression-free survival, overall survival and recurrence-free survival in the radiotherapy group compared to the control group [35]. According to the decision curves of validation group, 5- and 8-year net benefits were shown, displaying clear clinical value (Figure 9).

The process of using nomograms to predict 5- and 8-year OS and CSS was simple. First, we plotted vertical lines from clinicopathological factors to the points axis. When the total number of points was obtained, we drew vertical lines from total points to the prediction axes for 5- and 8-year OS and CSS. To a certain extent, prognosis is more accurately predicted using a nomogram than TNM staging. For instance, we used two T4N0M0 patients as example. Information of patient 1: 60 years old, female, White, grade I, surgery, radiation, T4N0M0. Information of patient 2: 45 years old, male, Black, grade III, non-surgery, non-radiation, T4N0M0. The prognosis of the above two patients was the same when using the TNM staging system. However, based on the nomograms model, 5-year OS was 47% and 32% for two patients. The 8-year OS was 33% and 20%, respectively. Moreover, the 5-year CSS was 54% and 26%, and the 8-year CSS was 47% and 19%, respectively. The above calculation and prediction based on the nomogram model can improve screening ability and make it possible to conduct the early intervention regarding controllable risk elements. Additionally, clinicians should pay attention to high-risk patients with multiple risk elements and develop the corresponding treatment plan and follow-up strategy.

Our research has both strengths and limitations. First, we established two reliable nomograms to provide assistance to surgeons. However, our research also has limitations. For example, other elements that may have influenced the prognoses of bladder cancer patients, such as body mass index [36], occupational hazards [37], genetic factors [38], chemotherapy [39], intravesical therapy [40], metabolic syndrome, waist circumference [7] and detrusor muscle thickening [6]. In addition, bladder cancer included transitional cell carcinoma (ca. 90%) and some rarer types, including squamous cell bladder cancer, adenocarcinoma, sarcoma, and small cell bladder cancer, etc. [41]. However, no information regarding various histologic subtypes of bladder cancer was documented in the SEER database. In future prospective studies, different histological subtypes need to be screened and included in the nomogram model. Different histological variants had an impact on the prognosis of bladder cancer [42]. Bladder urothelial carcinoma with histological variants were more likely to be diagnosed at advanced stage accompanying extravesical disease and metastasis [34]. However, the reality we need to recognize is that diagnosis of histological variants based on samples obtained by trans-urethral resection of bladder tumor (TURBt) is challenging. Analysis of TURBt specimens showed that only 39% of cases’ histological variants being subsequently confirmed at radical cystectomy [43]. Moreover, up to 44% of cases of histological variants were not identified or recorded by community pathologists [44]. Hence, collaborative efforts need to be made to improve diagnosis accuracy and understanding of these histological variants [34]. In addition to making the primary pathological diagnosis, the pathologist needs to determine whether various variants are combined. Moreover, chemotherapy can affect the progression of cancer and the regimen varies according to muscle infiltration condition. However, chemotherapy was usually performed outside of the hospital and the data were incomplete in public SEER database [45]. In addition, recurrence-free survival is also an important parameter for assessing prognosis, and the treatment applied after a recurrence can also influence OS and CSS. Because the above factors were not included in the SEER database, our nomogram models could not account for these characteristics. This is the common drawback of retrospective studies that researchers are unable to obtain some key factors from patient’s data, leading to clinical parameters selection bias reflecting patient prognosis. In the future, we plan to conduct multi-centered prospective research to incorporate more parameters into nomogram construction. Our nomogram can provide a reference for patients’ risk classification, survival prediction and clinician’s decision-making. Clinicians need to combine our nomogram prediction with patients’ symptoms, medical history, comorbidities, cancer progression, treatment and presence of histological variants or not to estimate specific individual prognosis outcomes empirically and comprehensively.

In conclusion, we conscientiously performed univariate and multivariate survival analyses and successfully established and validated two credible nomograms that could provide a reference for surgeons to utilize to tailor treatment plans and better evaluate prognoses. 

## Figures and Tables

**Table 1:** Clinicopathological data for patients.

Variables	Modeling Cohort (n = 10,478)		Validation Cohort (n = 10,379)	
	n	%	n	%
Age				
15–45	271	2.6	281	2.7
46–55	1033	9.9	954	9.2
56–65	2358	22.5	2366	22.8
66–75	3003	28.7	2940	28.3
76–85	2801	26.7	2855	27.5
85+	1012	9.7	983	9.5
Sex				
Male	7768	74.1	7816	75.3
Female	2710	25.9	2563	24.7
Race				
White	9234	88.1	9151	88.2
Black	741	7.1	747	7.2
Others	503	4.8	481	4.6
Grade				
I	852	8.1	806	7.8
II	2611	24.9	2706	26.1
III	7015	66.9	6867	66.2
Surgery				
Performed	10,145	96.8	9998	96.3
None	333	3.2	381	3.7
Radiation				
Yes	922	8.8	907	8.7
No	9556	91.2	9472	91.3
T stage				
T1	6037	57.6	5960	57.4
T2	2799	26.7	2757	26.6
T3	898	8.6	930	9.0
T4	744	7.1	732	7.1
N stage				
N0	9682	92.4	9563	92.1
N1	443	4.2	471	4.5
N2	336	3.2	322	3.1
N3	17	0.2	23	0.2
M stage				
M0	10,006	95.5	9897	95.4
M1	472	4.5	482	4.6

Abbreviations: Others: American Indian/AK Native, Asian/Pacific Islander. Grade I: Well differentiated. II: Moderately differentiated. III: Poorly differentiated.

**Table 2:** Survival analyses of OS in nomogram modeling cohort.

Variables	Uni-Variate Analysis			Multivariate Analysis	
	*p* Value			HR (95% CI)	*p* Value
Age		<0.001			<0.001
15–45				0.205 (0.163–0.257)	<0.001
46–55				0.199 (0.175–0.227)	<0.001
56–65				0.248 (0.224–0.273)	<0.001
66–75				0.345 (0.316–0.377)	<0.001
76–85				0.573 (0.526–0.623)	<0.001
85+				Reference	
Race		<0.001			<0.001
White				Reference	
Black				1.212 (1.094–1.343)	<0.001
Others				0.719 (0.621–0.832)	<0.001
Grade		<0.001			<0.001
I				0.720 (0.635–0.816)	<0.001
II				0.772 (0.717–0.831)	<0.001
III				Reference	
Surgery		<0.001			<0.001
Performed				Reference	
None				1.332 (1.148–1.546)	<0.001
Radiation		<0.001			<0.001
Yes				Reference	
No				0.775 (0.713–0.843)	<0.001
T stage		<0.001			<0.001
T1				0.330 (0.298–0.365)	<0.001
T2				0.637 (0.578–0.702)	<0.001
T3				0.725 (0.646–0.814)	<0.001
T4				Reference	
N stage		<0.001			<0.001
N0				0.381 (0.232–0.626)	<0.001
N1				0.599 (0.362–0.991)	0.046
N2				0.670 (0.404–1.112)	0.122
N3				Reference	
M stage		<0.001			<0.001
M0				0.341 (0.306–0.380)	<0.001
M1				Reference	

Abbreviation: Others: American Indian/AK Native, Asian/Pacific Islander. Grade I: Well differentiated. II: Moderately differentiated. III: Poorly differentiated.

**Table 3:** Survival analysis of CSS in nomogram modeling cohort.

Variables	Uni-Variate Analysis			Multivariate Analysis	
	*p* Value			HR (95% CI)	*p* Value
Age		<0.001			<0.001
15–45				0.353 (0.274–0.456)	<0.001
46–55				0.326 (0.279–0.381)	<0.001
56–65				0.363 (0.320–0.413)	<0.001
66–75				0.432 (0.383–0.487)	<0.001
76–85				0.639 (0.569–0.717)	<0.001
85+				Reference	
Sex		<0.001			0.001
Male				Reference	
Female				1.137 (1.053–1.228)	0.001
Race		<0.001			<0.001
White				Reference	
Black				1.243 (1.097–1.407)	0.001
Others				0.717 (0.593–0.867)	0.001
Grade		<0.001			<0.001
I				0.575 (0.475–0.697)	<0.001
II				0.676 (0.609–0.749)	<0.001
III				Reference	
Surgery		<0.001			<0.001
Performed				Reference	
None				1.538 (1.287–1.838)	<0.001
Radiation		<0.001			<0.001
Yes				Reference	
No				0.749 (0.680–0.827)	<0.001
T stage		<0.001			<0.001
T1				0.199 (0.176–0.225)	<0.001
T2				0.595 (0.533–0.663)	<0.001
T3				0.702 (0.617–0.799)	<0.001
T4				Reference	
N stage		<0.001			<0.001
N0				0.377 (0.226–0.630)	<0.001
N1				0.614 (0.365–1.034)	0.067
N2				0.700 (0.414–1.182)	0.182
N3				Reference	
M stage		<0.001			<0.001
M0				0.314 (0.279–0.353)	<0.001
M1				Reference	

Abbreviation: Others: American Indian/AK Native, Asian/Pacific Islander. Grade: I: Well differentiated. II: Moderately differentiated. III: Poorly differentiated.

## Data Availability

The datasets used and/or analyzed during the current study are available from the corresponding author on reasonable request.
